# The Mechanism of Tigecycline Resistance in *Acinetobacter baumannii* Revealed by Proteomic and Genomic Analysis

**DOI:** 10.3390/ijms24108652

**Published:** 2023-05-12

**Authors:** Cunwei Liu, Lei Wang, Ping Wang, Di Xiao, Qinghua Zou

**Affiliations:** 1 Department of Microbiology and Infectious Disease Center, School of Basic Medical Sciences, Peking University, Beijing 100191, China; 2 State Key Laboratory of Communicable Disease Prevention and Control, Collaborative Innovation Center for Diagnosis and Treatment of Infectious Diseases, National Institute for Communicable Disease Control and Prevention, Chinese Center for Disease Control and Prevention, Beijing 102206, China

**Keywords:** *Acinetobacter baumannii*, tigecycline resistance, proteome, whole genome sequencing, insertion sequence

## Abstract

The mechanism of tigecycline resistance in *A. baumannii* remains largely unclear. In this study, we selected a tigecycline-resistant and a tigecycline-susceptible strain from a tigecycline-susceptible and a resistant strain, respectively. Proteomic and genomic analyses were performed to elucidate the variations associated with tigecycline resistance. Our study showed proteins associated with efflux pump, biofilm formation, iron acquisition, stress response, and metabolic ability are upregulated in tigecycline resistant strains, and efflux pump should be the key mechanism for tigecycline resistance. By genomic analysis, we found several changes in the genome that can explain the increased level of efflux pump, including the loss of the global negative regulator *hns* in the plasmid and the disruption of the *hns* gene and *acrR* gene on the chromosome by the insertion of IS5. Collectively, we not only revealed the phenomenon that the efflux pump is mainly responsible for tigecycline resistance, but also highlighted the mechanism at the genomic level, which will help in understanding the resistance mechanism in detail and provide clues for the treatment of clinical multiple drug-resistant *A. baumannii*.

## 1. Introduction

Hospital-acquired infections caused by multi-antibiotic-resistant gram-negative bacteria have become an extremely serious problem in recent decades. In the late 1970s, due to the abuse of broad-spectrum antibiotics and the extensive use of antibiotics in agriculture and animal husbandry, the antibiotic resistance of *A. baumannii* increased dramatically. At present, *A. baumannii* has developed resistance to almost all commonly used antibiotics and has been listed as one of the six antimicrobial-resistant ESKAPE (*Enterococcus faecium*, *Staphylococcus aureus*, *Klebsiella pneumonia*, *Acinetobacter baumannii*, *Pseudomonas aeruginosa,* and *Enterobacter* species) pathogens by the World Health Organization [1]. Carbapenem is generally the conventional treatment for *A. baumannii* infection; however, outbreaks of infections caused by carbapenem-resistant strains have already been reported since the early 1990s [2], making polymyxin and tigecycline the last resort to treat multiple antibiotic-resistant strains of *A. baumannii* up until now.

Tigecycline, the third generation of tetracycline antibiotics [3], is a derivative of minocycline available to treat infections caused by tetracycline-resistant bacteria [4]. Tigecycline has strong in vitro biological activity against both gram-negative and gram-positive bacteria and was introduced into clinical treatment from 2005 to 2006 as the last resort of defense against multiple antibiotic-resistant bacteria [3]. Unfortunately, shortly after it was introduced into clinics, tigecycline-resistant *A. baumannii* appeared, and the incidence of infections caused by tigecycline-resistant strains has certainly increased in recent years [5].

At present, the mechanism of *A. baumannii* to tigecycline resistance is barely understood. Some studies using real-time reverse transcriptase PCR to correlate antimicrobial resistance with the expression of genes encoding efflux systems highlighted that overexpression of the AdeABC system was significantly associated with resistance to tigecycline [6,7,8]. Other studies also outlined the high incidence of resistance-nodulation-cell division (RND)-type transporters, mainly the AdeABC, AdeFGH, and AdeIJK efflux pumps overexpression in MDR *A. baumannii*, but other resistance mechanisms may also be implicated [9,10,11]. Wang et al. found that plasmids containing *tet(X3)* and *tet(X5)* which encode flavin-dependent monooxygenase, can mediate tigecycline resistance [12]. In addition, deletion or mutation of the *trm* gene encoding S-adenosyl-L-methionine-dependent methyltransferase and the *plsc* gene encoding 1-acyl-sn-glycero-3-phosphoacyltransferase can also result in increased tigecycline resistance [13,14], and deletion of the *arp* gene encoding peptidase C13 family can enhance cell membrane permeability and reduce bacterial resistance to tigecycline [15]. All these studies suggest that the mechanism of tigecycline resistance is complicated and that multiple antibiotic resistance mechanisms coexist. Tigecycline is still an attractive choice for *A. baumannii*, and further investigations are warranted so that effective treatment of MDR *A. baumannii* could be guided by validated in vitro data. In this study, we reversed the tigecycline-resistance in two strains by sub-minimum inhibitory concentration of tigecycline and high temperature, respectively, and comprehensively compared the proteomics and genomic differences of the strains before and after selection. We found variations both at the genomic and proteomic levels that were associated with tigecycline resistance. Our results will provide important clues for better understanding the mechanism of tigecycline resistance in *A. baumannii*.

## 2. Results

### 2.1. Selection of Tigecycline-Resistant and Tigecycline-Susceptible Strains from Tigecycline-Susceptible and Tigecycline-Resistant Strains, Respectively

To better understand the mechanism of tigecycline resistance, we intended to select a tigecycline-resistant strain from the parent tigecycline-susceptible strain and then find the changes both at the genome and proteome. We incubated a tigecycline-susceptible strain 17978S with successive sub-inhibitory concentrations of tigecycline, and a strain 17978R with a tigecycline-resistant phenotype was successfully selected. The MIC to tigecycline of 17978R increased to 128 μg/mL compared with 0.5 μg/mL of 17978S. For comparison, we also incubated a tigecycline-resistant strain A54R at 42 °C, and a strain A54S with a tigecycline-susceptible phenotype was selected. The MIC of A54S reduced to 1 μg/ mL compared with 8 μg/mL of A54R. All selected strains were sub-cultured for 15 generations (days) under non-tigecycline conditions, and the tigecycline MICs did not change after passaging, suggesting that the tigecycline resistance after selection is stable. It is worth noting that the selected tigecycline-susceptible strain A54S could still recover its resistance to tigecycline with a recovery rate of approximately 0.0000015 ± 0.0000007.

### 2.2. Proteomic Variations Associated with Tigecycline Resistance

**Figure 1:**
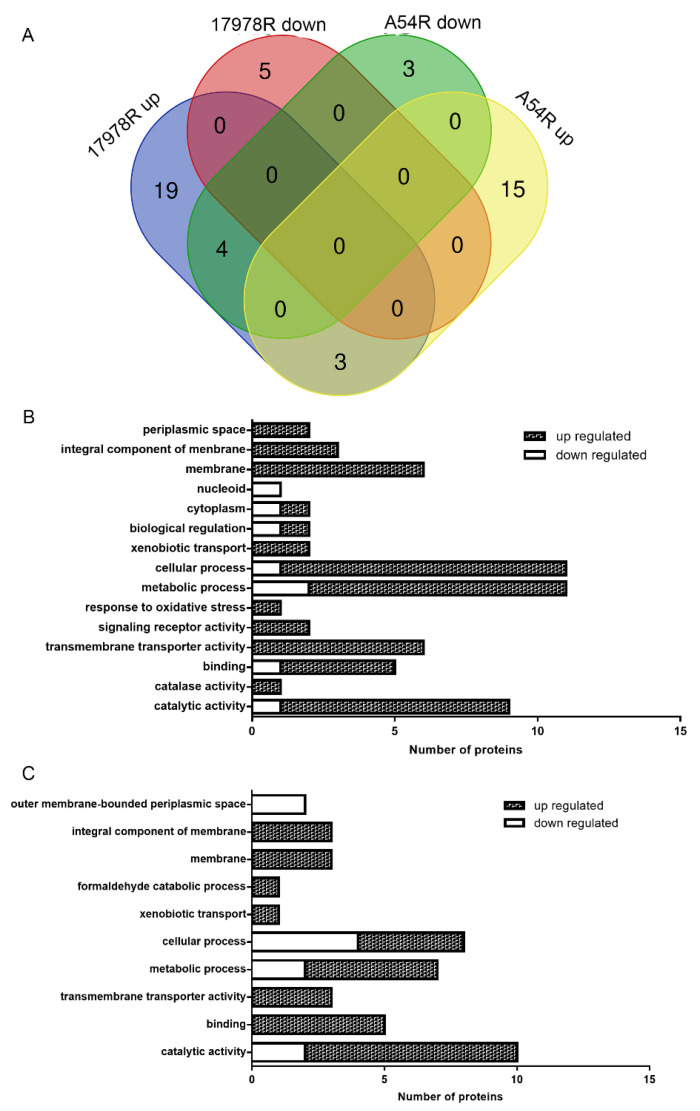
Venn diagram of differentially expressed proteins in 17978S, 17978R, A54R, and A54S, and GO classification of the differentially expressed proteins. (**A**) Venn diagram; (**B**,**C**) show the number of differential proteins of 17978 S/R (**B**) and A54 R/S (**C**) in each GO type, respectively. Up-regulation means that the protein expressed in the resistant strain R has a foldchange ≥2 compared to that of the susceptible strain S, and down-regulation means that the expression of the protein in the resistant strain R has a foldchange ≤1/2 compared to that of the susceptible strain S.

We first focused our attention on tigecycline resistance-related proteins in the two pairs of strains. Thirty-one different proteins were identified between 17978S and 17978R, of which twenty-six were up-regulated and five were down-regulated in 17978R. Twenty-five differential proteins were identified between A54R and A54S, of which eighteen were up-regulated and seven were down-regulated in A54R (Figure 1A, Table S1). The up-regulated proteins in 17978R contain the efflux pump transporter, type I pili-related proteins CsuA/B, CsuC, CsuE, catalase, glutamate dehydrogenase, integrase, and TonB-dependent ferroportin. The down-regulated proteins in 17978R contain HNS domain-containing proteins and LysM domain-containing proteins. GO analysis showed that the up-regulated proteins are mainly involved in metabolic processes, cellular processes, and catalytic processes, while the down-regulated proteins belong to the nucleoid classification (Figure 1B). The up-regulated proteins in A54R contain the efflux pump membrane transporter, formaldehyde dehydrogenase, and glucose-arabinose dehydrogenase. Unexpectedly, the up-regulated proteins CsuA/B putative secreted protein, CsuC, signal peptide, which were up-regulated in 17978R, were down-regulated in A54R (Table S1). GO classification showed that the up-regulated proteins in A54R are involved in the classification of catalytic function and membrane, while those down-regulated in A54R function in cellular process and the outer membrane periplasm (Figure 1C). It is worth noting that there are three proteins—efflux pump membrane transporter, the efflux transporter periplasmic adaptor subunit, and the SDR family oxidoreductase—up-regulated in both the tigecycline-resistant strains 17978R and A54R (Figure 1A, Table S1).

### 2.3. Efflux Pump Plays Important Roles in Tigecycline Resistance

Since efflux pump-related proteins were up-regulated both in the tigecycline-resistant strains 17978R and A54R, and previous studies also showed efflux pumps play important roles in antibiotic resistance [16], we tried to see whether the efflux pumps play roles in the tigecycline resistance in the two pairs of strains. We incubated the strains with efflux pump inhibitors PAβN or CCCP and found that in the presence of PAβN and CCCP, the MICs of the selected tigecycline-resistant strain 17978R decreased by 2 folds and 8 folds, respectively (Table 1), and no difference were found in A54R, A54S and 17978S. However, although the MIC of 17978R decreased in the presence of the efflux pump inhibitor, it did not return to the level of 17978S, suggesting that although the efflux pumps play important roles in the tigecycline resistance, there should be other mechanisms involved.

### 2.4. Genomic Variation of Tigecycline-Susceptible and Tigecycline-Resistant Strains before and after Selection

In order to further explore the tigecycline resistance mechanism, we conducted third-generation whole genome sequencing analysis on the two pairs of strains to study the genetic differences before and after selection. The information on the whole genome sequencing is shown in Table S2. The numbers of the base pairs of 17978S, 17978R, A54R, and A54S were 4,075,596 bp, 3,917,421 bp, 4,236,314 bp, and 4,108,976 bp, respectively. The numbers of the coding genes were 3,933, 3,762, 4,159, and 4,024, respectively. Apparently, the selected strains 17978R and A54S have fewer base pairs than the initial strains 17978S and A54S. We then analyzed the chromosomes and plasmids in the strains to explore the differences.

#### 2.4.1. Plasmid Variation before and after Selection

**Figure 2:**
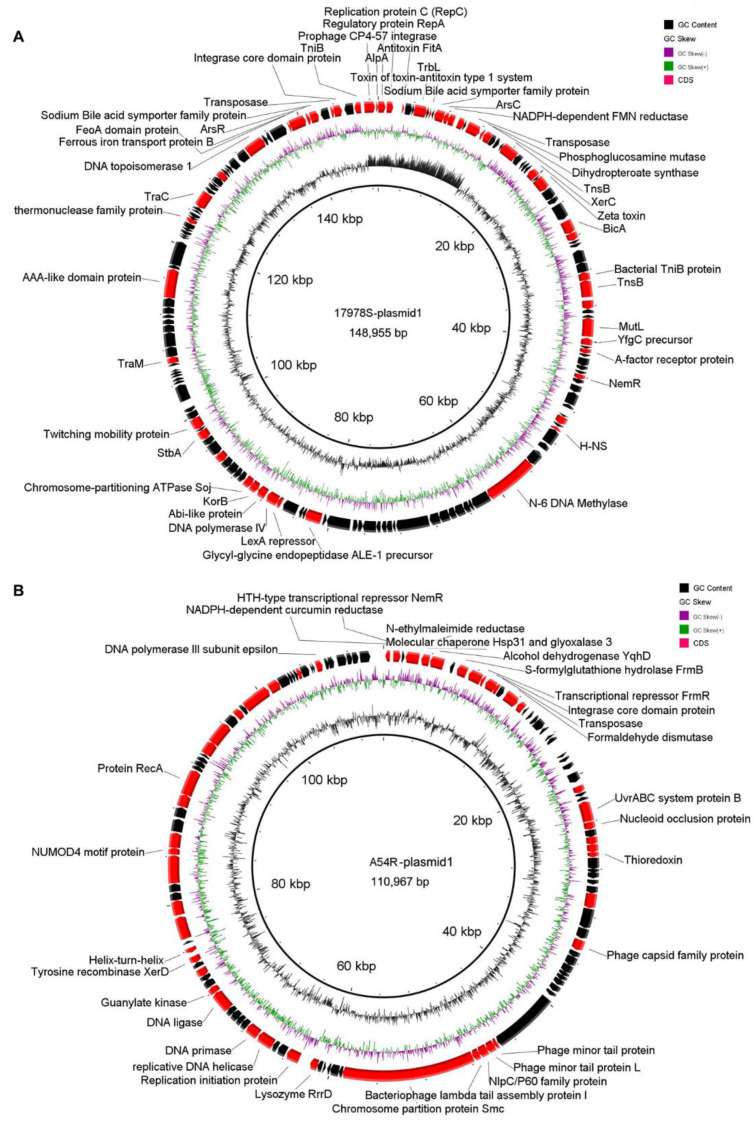
Coding genes on 17978S-plasmid 1 and A54R-plasmid 1 were lost during selection. (**A**) 17978R-plasmid 1 BRIG circle diagram, from outside to inside, gene-encoding protein (black represents hypothetical protein, red represent coding genes), GC skew, and GC content; (**B**) A54R-plasmid 1 BRIG circle diagram, from outside to inside, gene-encoding protein (black represents hypothetical protein, red represent coding genes), GC skew, and GC content.

17978S and A54R both contain two plasmids, with sizes of 148,955 bp (17978S-plasmid 1) and 24,822 bp (17978S-plasmid 2) in 17978S, and 110,967 bp (A54R-plasmid 1) and 69,098 bp (A54R-plasmid 2), respectively. Compared with 17978S, the large plasmid (17978S-plasmid 1) was lost in the selected strain 17978R. The plasmid 17978S-plasmid 1 contains 156 encoding genes, 105 of them encoding hypothetical proteins; the remaining genes encode proteins involved in plasmid conjugation and replication, growth metabolism, virulence, and transposition. It is worth noting that a *hns* gene was found in this plasmid. No genes directly related to antibiotic resistance were found (Figure 2A). Compared with A54R, the large plasmid (A54R-plasmid 1) with a size of 110,967 bp was lost in the selected strain A54S (Figure 2B). The A54R-plasmid 1 shares high similarity to the plasmid pXH386 of *A. baumannii* XH386 (query cover = 100%, percent identity = 100%). XH386 is an MDR strain isolated from a pediatric hospital in China [17], and its ST type belongs to ST208, showing resistance to most antibiotics, such as tetracycline and levofloxacin. The genes on this plasmid encode phage-related proteins, enzymes, including S-formyl glutathione hydrolase, S-(hydroxymethyl) glutathione dehydrogenase, and transporters, including phosphonate/organophosphate ester transfer proteins, IS and transposases, transcriptional regulators, including FrmR [18], DNA synthesis, and repair-related proteins (Figure 2B). These proteins are mainly involved in growth, metabolism, and regulation, and their functions are similar to those on 17978S-plasmid 1.

It was reported that plasmid-mediated resistance to tigecycline is usually due to the flavin-dependent mono-oxygenase-coding gene *tet(X)* [12]. We analyzed the plasmids and found no *tet(X)* gene in all the plasmids in 17978R and A54R, suggesting that the tigecycline resistance is not mediated by *tet(X)*-containing plasmids in A54R and 17978R.

#### 2.4.2. Chromosome Variation before and after Selection

**Figure 3:**
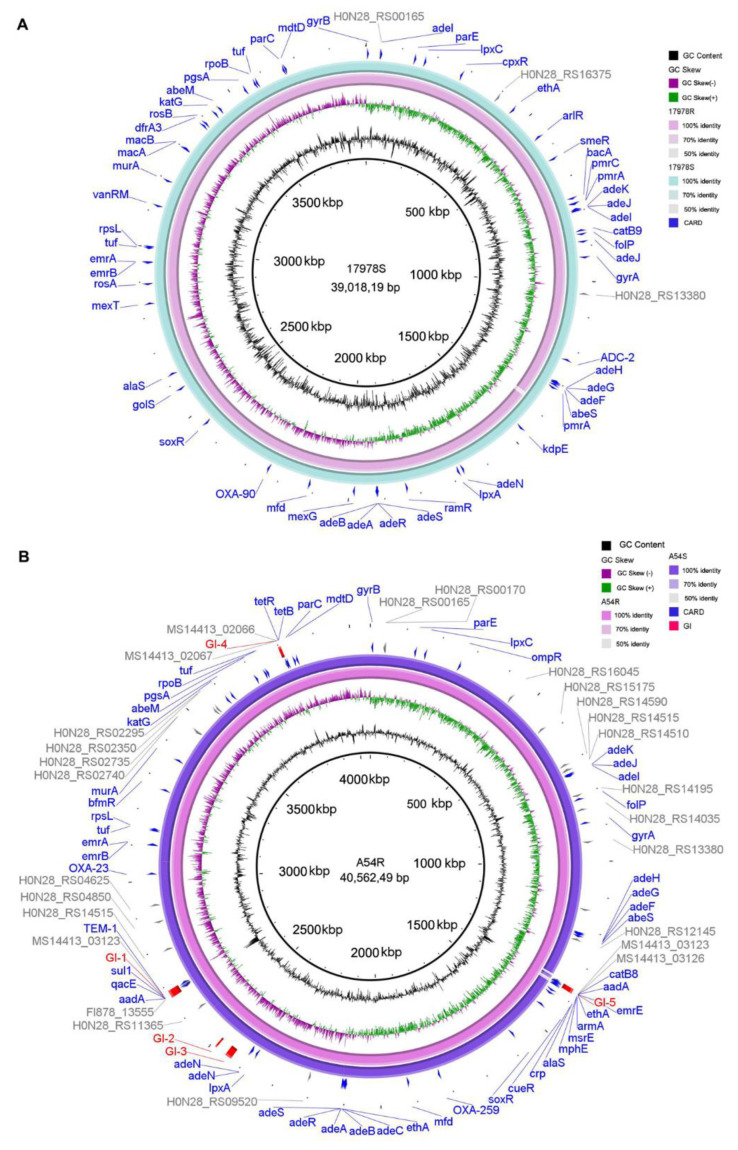
Comparisons of the chromosomes of 17978S/R (A) and A54R/S (**B**). (**A**) The 17978S/R BRIG circle diagram, from outside to inside, CARD, 1978S, 178978R, GC skew, and GC content; (**B**) A54R/S BRIG circle diagram, from outside to inside, GI, CARD, A54S, A54R, GC skew, and GC content.

Comparative genomic analysis with MAUVE showed distinct variations between 17978S, 17978R, A54R, and A54S. The genome has high collinearity between 17978S and 17978R, and no large sequence rearrangements were found (Figure S1A). In contrast, compared with A54S, there is a large fragment (approximately 1500 kb) inverted in the chromosomal region of A54R (Figure S1B). Further analysis showed that compared with 17978S, there is a large deletion at 1,375,662 to 1,387,876 bp in 17978R (Figure 3A), and A54S had a deletion of ~25 kb at 1,357,422 to 1,383,342 bp, which was localized at the genomic island GI-5 compared with A54R (Figure 3B). The coding genes in the deletions are listed in Table S3. It is worth noting that some antibiotic resistance genes were found in these deletions; in 17978S, the genes contain *acrR*, which encodes a transcriptional regulator for AcrAB. In A54R, the genes contain *armA*, *msrE,* and *mphG*, which are associated with aminoglycoside and macrolide antibiotic resistance.

Next, we analyzed the single nucleotide variations (SNV) in the strains (Table S4). There are 20 SNVs in 17978S/R. Seven out of them occur in coding genes, among which, ACX60_03215 encodes a SAM-dependent methyltransferase, and previous studies showed that mutation of this gene reduced the sensitivity of *A. baumannii* ATCC 19606 to tigecycline, minocycline, and doxycycline [13]. In this study, the SNV changed the encoded amino acid from lysine to cysteine. There are 102 SNVs in A54R/S, among which 38 lie in non-coding regions. The coding genes include *ata*, *ahpF,* and *tuf*. Ata is an important virulence factor for many gram-negative bacteria, which can help mediate adhesion and invasion, induce apoptosis, and cause host diseases.

### 2.5. Variation of Antibiotic Resistance Genes, Virulence Factors, and Genomic Islands

In addition, we compared the antibiotic resistance genes, virulence factors, and genomic islands in the two pairs of strains. Results were shown in Table S5. Additionally, 17978S has one more CARD gene *sul2* than 17978R, which is involved in the resistance to sulfonamide antibiotics [19]. This gene is located in 17978S-plasmid 1 and is lost in 17978R (Figure 3A). A54R has nine more CARD genes than A54S, eight of them *armA*, *msrE*, *mphG*, *sul2*, and *emrE* are located on the genome island GI-5 of A54R (Figure 3A). The GI-5 is lost when A54R is selected into A54S. The other gene encodes β-Lactamase *bla*_OXA-23_, which is involved in the resistance to carbapenems. The number of virulence genes in the selected strains 17978R and A54S decreased 2~6 compared with the pre-selected strain. There is no difference in the number of genome islands in 17978S/R, but after A54R is selected into A54S, GI-5 is lost. It contains multi-drug resistance related genes listed above.

### 2.6. Variation of IS Elements before and after Selection

**Figure 4:**
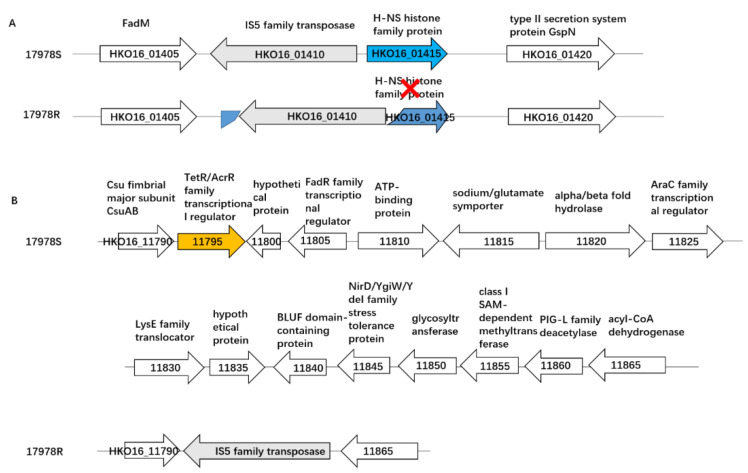
Variation of IS5 in the tigecycline-selected strain 17978R compared with 17978S. (**A**) The insertion of IS5 in the *hns* gene in 17978R; (**B**) the insertion of IS5 resulted in the deletion of 15 genes, including *acrR* in 17978R. The genes were named based on the locus tag of *A. baumannii* strain ATCC17978 with the accession number CP053098 in the GeneBank Genome database. The red cross means the disruption of *hns* gene by the insertion of IS5.

IS transposition can affect the antibiotic resistance and virulence of bacteria by changing gene expression [20]. We used ISEscan [21] to analyze the distribution of IS in the strains and found that in 17978S, there is a gene encoding the IS5 family transposase adjacent to the *hns* gene, while it translocated into the *hns* gene in the tigecycline-selected strain 17978R, resulting in the disruption of *hns* (Figure 4A). At the same time, we found an IS5 insertion between the genes HKO16_11790 and HKO16_11865, leading to the loss of 15 coding genes, including the gene *acrR* (Figure 4B). Previous studies have shown that H-NS is a global regulator and is involved in the regulation of antibiotic resistance [22], natural transformation [23], and virulence [24], and AcrR is an efflux pump regulator. We then tested whether the insertion of the IS element in the two regions could influence tigecycline sensitivity.

### 2.7. Deletion of Hns Decreased Tigecycline Sensitivity in 17978S

**Figure 5:**
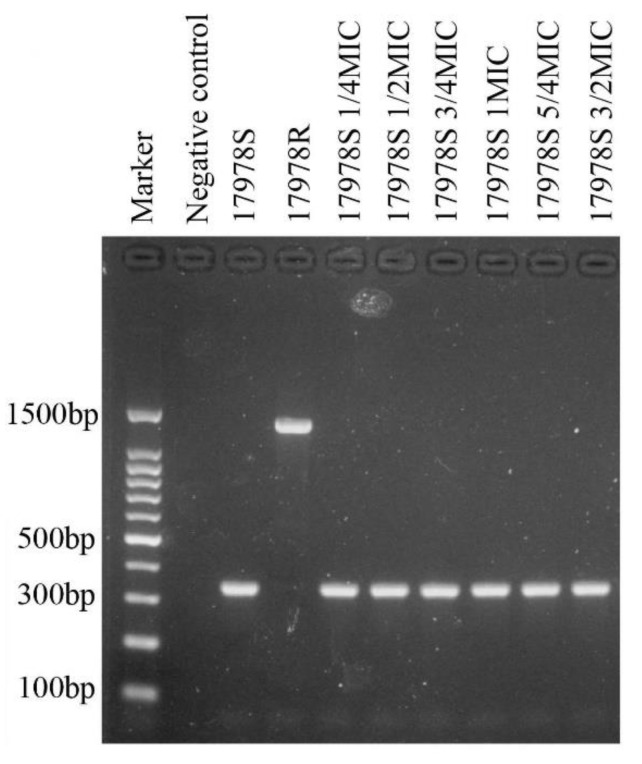
PCR results of the *hns* gene in sub-MIC-selected strains of 17978R. IS5 inserted into the *hns* of 17978R (lane 4). In the repeated experiment, no insertion was found at any time point (lane 5–10).

In 17978S, there are two *hns* genes. One is located on the plasmid, which was lost along with the 17978 plasmid-1 during the selection of 17978R; the other was disrupted by IS insertion. Therefore, 17978R completely lost the function of expressing H-NS. To confirm the role of H-NS in tigecycline resistance, we first used the pTrc99A plasmid to complement the *hns* gene for 17978R. The results showed that, compared with 17978R, the MIC to tigecycline of the complemented strain increased from 128 ng/μL to 64 ng/μL. We further constructed the 17978S *hns* deletion mutant. As expected, the mutant had improved tigecycline resistance (MIC: 0.5 ng/μL → 1 ng/μL), suggesting the involvement of H-NS in regulating the tigecycline resistance of *A. baumannii*. Then, we sought to figure out whether the insertion of IS5 in the *hns* gene was a random or fixed event when *A. baumannii* was put under the pressure of sub-MIC tigecycline. We performed the selection experiment again and checked the *hns* gene in the strains during the selection process and found no IS5 insertion into the *hns* gene at any time point during the selection process (Figure 5), indicating that the insertion of IS5 altering the *hns* gene occurred randomly under the pressure of sub-MIC tigecycline.

### 2.8. Proteomic Analysis of 17978S and Hns-Deleted Strains

Since H-NS often acts as a global regulator, we performed iTRAQ proteomic analysis on the *hns* gene deletion strains 17978S△*hns* and 17978S to find proteins that were regulated by H-NS and related to tigecycline resistance. A total of 127 differentially expressed proteins were found. Among them, 90 proteins were up-regulated in the *hns* deletion mutant, and only 37 proteins were down-regulated (Table S6), which is consistent with previous reports [25] that H-NS is mainly a negative regulator. The down-regulated proteins include metabolic enzymes such as oxidoreductase, amino acid transferase, etc., transcriptional regulators, toxin-related proteins, etc. The up-regulated proteins include the TonB-dependent siderophore receptor [26], Ata autotransporter adhesin [27], manganese transporter, catalase, etc. These up-regulated proteins are mainly involved in the regulation of bacterial activities and resistance to external adverse events.

### 2.9. Complement of AcrR to 17978R Reduces Tigecycline Resistance

Bacterial TetR/AcrR family transcriptional repressors are often involved in sensing the dynamic changes of the bacterial surrounding environment and in the regulation of various genes [28]. The regulator AcrR is involved in regulating the antibiotic resistance of bacteria by negatively regulating the expression of the efflux pump system AcrAB. Subhadra et al. [29] found that, compared with the wild-type strain, the expression of the genes *acrA* and *acrB* was increased in the *acrR* deletion mutant, confirming that *acrR* acts as a repressor of the *acrAB* operon. In this study, when the *acrR* was complemented in 17978R, we found that the MIC to tigecycline decreased from 128 ng/μL to 64 ng/μL, indicating the negative regulation of the *acrR* to tigecycline resistance in *A. baumannii*.

## 3. Discussion

The extreme resistance to a wide variety of antibiotics, especially the last resort antibiotics, of *A. baumannii* has posed great challenges to clinical treatment. In this study, we investigated the mechanism of *A. baumannii* to tigecycline resistance by comprehensively analyzing proteomics and genomic variations in tigecycline-selected strains. By comparative proteomics, we found efflux pump related proteins AdeB and AdeA were up-regulated in both tigecycline-resistant strains 17978R and A54R. The up-regulation of the AcrAB efflux system was also found in 17978R. This is consistent with previous studies [6,7,8,30] which found that AdeABC and AcrAB-TolC efflux systems were overexpressed in tigecycline-resistant strains. When blocked by efflux pump inhibitors, we found that PAβN and CCCP cannot completely reverse the tigecycline resistance, suggesting that although the efflux pump is one of the major factors affecting tigecycline resistance, it is not the only mechanism responsible for tigecycline resistance in these strains.

Csu fimbriae are adhesion organs for many gram-negative bacteria [31] and play important roles in the initial steps of biofilm formation [32,33,34]. Both the CUS (chaperon–usher secretion) operator system and the surface antigen protein A are related to the formation of biofilms [35,36]. In this study, these biofilm formation-related proteins CsuA/B, CsuC, CsuE were all up-regulated in 17978R, but down-regulated in A54R, which was consistent with the results of the biofilm-forming ability of the two strains [37]. The relationship between the biofilm-forming ability and antibiotic resistance is still controversial. We speculate that the increased biofilm forming ability in the 17978R by up-regulation of Csu may aid it in acquiring tigecycline resistance in a short time.

Plasmids are important elements mediating the acquisition and loss of genes. Weber et al. [38] found that *A.baumannii* MDR clinical strains had a large plasmid carrying the negative regulator of the type VI secretion system (T6SS). The loss of this large plasmid can activate T6SS, but also can reduce bacterial antibiotic resistance. In this study, both 17978S and A54R lost a large plasmid during the selection process. However, no antibiotic related genes were found in the plasmids. We transformed the plasmid of A54R into A54S or *E. coli* DH5α but failed to obtain tigecycline-resistant transformants, at the same time, A54S has a low probability of recovering its resistance, both suggesting that the plasmids in A54R are probably not the element that directly mediates tigecycline resistance. The lost plasmids encode a variety of proteins, including proteins related to plasmid conjugation and replication, toxin-related proteins, metabolism-related proteins, and DNA-related active proteins. These suggested that to adapt to the sub-MIC tigecycline environment or high temperature, *A. baumannii* has to discard genes that are not necessary for survival to regulate their basal life activities and focus on resisting external pressure.

By whole genome analysis, we found that the chromosome of 17978R selected by sub-MIC has high collinearity with 17978S, and there were no large fragment changes such as translocations, while in A54R/S a large fragment inversion appeared. This suggests that the selection pressure of sub-MIC selection on the genome of the strain is relatively mild, and the high temperature selection will make the strain adjust its genome to adapt to this strong survival pressure, so as to survive better. At the genomic level, we found *A. baumannii* may acquire tigecycline resistance by single nucleotide variation on some genes, such as ACX60_03215 which encodes a SAM-dependent methyltransferase. At the same time, both in 17978S and A54R, we found large fragment deletions that contain multi-drug resistance-related genes, suggesting that horizontal gene transfer should play an important role in the tigecycline resistance of *A.baumannii*.

Previous studies [39] have shown that IS transposition can be affected by external environment, and play key roles in genome evolution. Based on whole genome sequence analysis, this study found that the number of IS elements in *A. baumannii* was altered under sub-MIC tigecycline stress. IS elements can alter the antibiotic resistance of bacteria by inserting into the upstream of the carbapenemase and cephalosporinase resistance genes and thus increasing the expression of these genes, resulting in higher levels of antibiotic resistance properties [40,41,42,43]. In this study, we were surprised to see that under sub-MIC tigecycline, IS5 can translocate into *hns* in 17978R, and confirmed that this phenomenon occurs randomly, which is conducive to the random mutation of bacteria and helps the development of biodiversity.

We also found that the expression of H-NS was significantly down-regulated in 17978R, and genomics analysis revealed that the cause was the loss of the plasmid and disruption of *hns* gene by IS sequences in 17978R. H-NS has been shown to be associated with bacterial resistance to a variety of antibiotics [44,45]. It can also alleviate VIM-2 and SPM-1 selected stress, thereby increasing carbapenem resistance [40]. The ability to acquire metal nutrients such as iron, manganese, and zinc is crucial for bacterial infection of the host [46]. Free iron is a limited micronutrient in the host, and it is usually closely combined with biomolecules such as hemoglobin. The iron acquisition system is an important factor for bacterial pathogenesis [47]. In our study, it was found that in 17978R, PfeA, TonB Dependent siderophore receptors, and outer membrane receptor proteins are all up-regulated. Interestingly, we found the expression of ABC transporter family proteins and TonB-dependent siderophores receptors were both increased in the *hns* deletion mutant, suggesting the regulation of TonB-dependent siderophores by H-NS. A study by Zhao et al. indicated that drug efflux may require a TonB or TonB-like protein for the operation of the outer membrane components and influence the multi-drug resistance of *P. aeruginosa* [48]. Therefore, it is possible that, for 17978R, it can acquire resistance to tigecycline by IS insertion into *hns* or loss of plasmid containing *hns*, and thus increase the expression of TonB, then results in increased expression of the efflux pump.

In addition, the insertion of IS elements also caused the loss of *acrR*. As an important regulator of the AcrAB efflux pump, AcrR can also be involved in regulating the motility, biofilm formation and virulence of *Acinetobacter* [29]. Research [49] found that the quorum-sensing regulator AnoR can participate in the transcriptional activation of AcrAB, and there is an AcrR-binding sequence in the promoter region of QS genes such as *anoR*, which can specifically bind to it, thereby regulating the transcription of QS genes and indirectly regulating the expression of the pump AcrAB. As a repressor of the efflux pump AcrAB [29], AcrR can inhibit the expression of the efflux pump, and deletion of AcrR will increase the expression of AcrAB, thereby promoting the efflux of drugs and reducing the sensitivity of the strain. In addition, previous studies [50] have shown that AcrR involved in the regulation of bacterial resistance by reducing exercise capacity and biofilm formation capacity. As indicated above, in this study, the biofilm formation ability of 17978R increased, whether the loss of AcrR plays a role needs further investigation.

**Figure 6:**
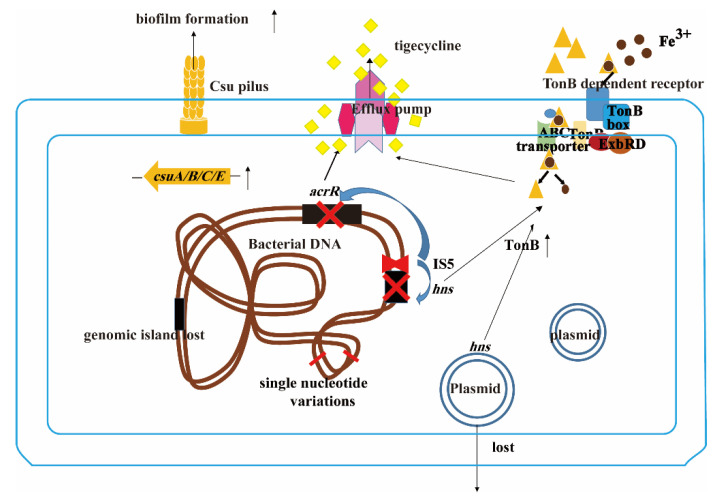
A proposed scheme used by *A. baumannii* under sub-MIC tigecycline. IS5 can insert into *hns* and *acrR* gene, plasmid containing *hns* can lost, which can lead to the up-regulation of efflux pump. At the same time single nucleotide variations, genomic island lost can also occur. The red cross means the deletion of *hns* and *acrR* gene by the insertion of IS5.

In conclusion, in this study, we found a variety of mechanisms related to tigecycline resistance. The overwhelming data indicated that the mechanism of tigecycline resistance is complex. However, we still found a correlation between these results. Our study showed efflux pump is up-regulated in tigecycline-resistant strains and is the key mechanism for tigecycline resistance. By genomics analysis, we found several changes in the genome, which can explain this phenomenon (Figure 6), including the loss of the global negative regulator *hns* in the plasmid and the disruption of the *hns* gene and *acrR* gene on the chromosome by the insertion of IS5. Loss of *hns* can result in increased expression level of TonB, which can increase the expression level of efflux pump. Previous studies have found some relationship between efflux pump and tigecycline resistance, however, no studies revealed the mechanism behind it. Taken together, the tigecycline resistance mechanism of *A. baumannii* is complex and we not only revealed the phenomenon that efflux pump is mainly responsible for tigecycline resistance, but also highlighted the mechanism at the genomic level. To our knowledge, this is the first study elaborating this issue from both proteomics and genomics analysis.

## 4. Materials and Methods

### 4.1. Bacteria Strains and Selection of Tigecycline Resistance Varied Strains

Tigecycline-susceptible strain ATCC 17978 (named 17978S in this study) was incubated with sub-MIC tigecycline and selected for tigecycline-resistant strain 17978R according to a previous study [51]. Briefly, the MIC of 17978S was tested using broth dilution method, then the strain was cultured overnight and transferred with a ratio of 1:100 to fresh MH broth containing 1/4 MIC of tigecycline. After 24 h, it was transferred to fresh MH broth containing 1/2 MIC tigecycline, then to 3/4 MIC, 1 MIC, and 3/2 MIC of tigecycline accordingly. If growth cessation were observed at some time point, the concentration of tigecycline was reduced to one half for cultivation. When growth arrived at 3/2 MIC, the MIC of the bacteria was tested, and the strain was named as 17978R. The tigecycline-resistant strain A54R was incubated and selected for tigecycline-susceptible strain A54S by high temperature. Briefly, a single colony of A54R was picked up and cultured in LB broth overnight for 24 h, and then sub-cultured every 24 h at 42 °C for 18 days. The final MIC was confirmed ≤2 μg/mL and the strain was named as A54S.

### 4.2. Stability of the Antibiotic Resistance of the Selected Strains

The stability test for strain’s tigecycline resistance was performed with broth dilution method. All the selected tigecycline-resistant and -susceptible strains were cultured in LB broth without antibiotics at 37 °C and sub-cultured in fresh broth every 24 h for 15 days. The MIC before and after passage was compared to see whether the strains have a stable tigecycline resistance phenotype. At the same time, the selected strain A54S was cultured on LB solid medium without antibiotics and LB solid medium containing 8 μg/mL tigecycline, the number of colonies were compared to test the recovery rate of tigecycline resistance.

### 4.3. Protein Sample Preparation

*A. baumannii* 17978S, 17978R, A54R, and A54S were grown to an optical density (OD_600_) of approximately 0.8 and then harvested using centrifugation at 8000× *g* for 10 min at 4 °C. The pellets were put into liquid nitrogen and grinded fully, and then suspended by phenol extraction solution with 1mM PMSF (Amresco, Washington, DC, USA) and sonicated on ice. An equal volume of phenol-Tris-HCl (pH7.8) saturated solution were added and mixed up for 30 min at 4 °C, then the supernatants were collected by centrifugation at 4 °C, 7100× *g* for 10 min. Five times the volume of pre-cooled 0.1 M ammonium acetate-methanol solution were added and precipitated overnight at −20 °C. The precipitates were washed with pre-cooled methanol and acetone and collected by centrifugation at 4 °C, 12,000× *g* for 10 min and dried at room temperature, then dissolved in lysis solution at room temperature for 3 h and the concentration was determined by using a BCA Protein Assay Kit (Thermo, Rockford, IL, USA).

### 4.4. Protein Digestion, iTRAQ Labeling and LC-MS/MS

Proteins from each sample (100 μg) were processed and subjected to isobaric tags for relative and absolute quantitation (iTRAQ) labeling using the AB SCIEX iTRAQ Regents Multiplex Kit (ABSCIEX, Shanghai, China) according to the manufacturer’s instructions. Briefly, proteins were first reduced and alkylated with DTT (Sangon, Shanghai, China) and iodoacetamide (Sangon, Shanghai, China), then digested with trypsin Trypsin-TPCK (Hualishi, Beijing, China) at 37 °C overnight. The digested samples were lyophilized and reconstituted with 100 mM TEAB (Sangon, Shanghai, China) and labeled with iTRAQ regent at room temperature for 2 h.

The iTRAQ-labeled peptides were reconstituted and separated using reverse-phase 1100 HPLC (Agilent, Palo Alto, CA, USA) using an Agilent Zorbax Extend-C18 column (2.1 × 150 mm, 5 μm) (Agilent, Palo Alto, CA, USA). In brief, an iTRAQ-labeled peptide mixture was eluted with ACN-H_2_O (2:98, *v*/*v*) with a gradient of 0~8min, 98% A; 8~8.01 min, 98~95% A; 8.01~48 min, 95~75% A; 48~60 min, 75~60% A; 60~60.01 min, 60~10% A; 60.01~70 min, 10% A; 70~70.01 min, 10~98% A; 70.01~75 min, 98% A. The eluents of 8~60 min were collected every minute, freeze dried and saved for mass spectrometry. The Q Exactive HF mass spectrometer (Thermo Scientific, Waltham, MA, USA) was used in combination with the EASY-nLC 1200 LC system (Thermo Scientific, Waltham, MA, USA) for the separation and identification of proteomes. The sample was loaded into the precolumn Acclaim PepMap 100,100 μm × 2 cm RP-C18 (Thermo Scientific, Waltham, MA, USA) at a flow rate of 300 nL/min, and then separated by the Acclaim Pepmap RSLC column, 75 μm × 15 cm RP-C18 (Thermo Scientific, Waltham, MA, USA). The quality resolution of the primary MS is 60,000, the automatic gain control value is 3e6, and the maximum injection time is 50 ms; the MS scanning is set as the full scanning charge mass ratio m/z range of 350–1500, and MS/MS scanning is performed for 20 of the highest peaks with resolution 15,000, automatic gain control 2e5, maximum ion injection time 40 ms, and the dynamic exclusion time 30 s. The data was deposited in ProteomeXchange with accession number PXD034784.

### 4.5. Downstream Processing and Analysis of Proteomics Data

MS/MS spectra were searched using Proteome Discover2.4 (Thermo Scientific, Waltham, MA, USA) software. Searches of protein identification were performed using the following criteria: static modification: iTRAQ (N-term, K, Y), carbamidomethyl(C); dynamic modification: oxidation (M), Acetyl(N-term); digestion: trypsin; instrument: Q Exactive HF; MS1 tolerance: 10 ppm; MS2 tolerance: 0.02Da; missed cleavages:2. The credible proteins were screened according to the criteria of score sequence HT > 0 and unique peptide ≥1, and blank value was removed. Proteins with foldchange ≥2 or foldchange ≤1/2 and *p* < 0.05 were considered to be differentially expressed. For the obtained differential proteins, NCBI BLAST+ and Uniprot databases were used for GO enrichment analysis (statistically significant differences in GO terms were defined by *p* < 0.05). 

### 4.6. Efflux Pump Inhibition Experiment

Efflux pump inhibitor PAβN (TargetMol, Shanghai, China) and CCCP (TargetMol, Shanghai, China) were used for the efflux pump inhibition experiment. The MICs of the bacteria were tested by either the dilution method in cation-regulated MH liquid medium containing 50 mg/mL PAβN or 10 mg/mL CCCP. The efflux pump inhibitory phenotype was considered positive when the MIC of a certain strain decreased by more than or equal to two folds in the presence of efflux pump inhibitor.

### 4.7. Whole Genome Sequencing

Total genomic DNA was extracted from overnight cultures using the Bacterial DNA Isolation Kit (Foregene, Beijing, China) following the manufacturer’s instructions, followed by genomic DNA sequencing using the Illumina Hiseq™ sequencer (Illumina, San Diego, CA, USA) and PacBio RSII system (PacBio, Menlo Park, CA, USA). FastQC (Version 0.11.2) was used for quality control. Adaptor sequences were trimmed and removed using Trimmomatic. The short-read sequence was assembled using SPAdes v3.10.0 [52]. GapFiller [53] was used to complement the GAP of the spliced contig. PrInSeS-G [54] was used for sequence correction. Prokka was used to predict gene elements: genes, tRNA, rRNA, etc. RepeatMasker was used to identify repetitive sequences in the genome. NCBI Blast+ [55] was used to compare the gene coding sequence with CDD, KOG, COG, NR, NT, PFAM, Swissprot, and TrEMBL to obtain its functional annotation information. Virulence factors, antibiotic resistance genes were obtained by using NCBI Blast+ to compare the gene/protein sequence with VFDB and CARD to obtain the functional annotation information. BRIG (http://sourceforge.net/projects/brig, assessed on 2 March 2022) and Mauve (https://sourceforge.net/projects/mauve/, assessed on 20 March 2022) were used for genome linear analysis. IS elements were predicted using ISEScan [21] with default parameters. The sequences were deposited in GenBank with BioProject ID PRJNA850273.

### 4.8. Gene Knockout and Complement

Gene knockouts were performed as described previously [56]. Briefly, a 1 kb fragment containing the upstream and downstream of the target gene was amplified by PCR, and then ligated to the pMo130-TelR plasmid. The re-constructed plasmid was transformed into 17978S and plated onto LB plates with 10% sucrose to screen for gene deletion mutants. pTrc99A plasmid was used for gene complement.

## Figures and Tables

**Table 1:** MICs (μg/mL) of the strains to tigecycline with and without an efflux pump inhibitor.

Stains	Without Inhibitor	+PAβN	+CCCP
17978S	0.5	0.5	0.5
17978R	128	64	16
A54S	8	8	8
A54R	1	1	1

## Data Availability

The proteomics data was deposited in ProteomeXchange with accession number PXD034784. The complete genome sequence data were deposited in GenBank with BioProject ID PRJNA850273.

## Supplementary Materials

The following supporting information can be downloaded at: https://www.mdpi.com/article/10.3390/ijms24108652/s1.
