# Supplementary material for: The Mechanism of Tigecycline Resistance in Acinetobacter baumannii Revealed by Proteomic and Genomic Analysis
Source: Int J Mol Sci. 2023 May 12;24(10):8652. doi: 10.3390/ijms24108652 (PMC10218405; doi:10.3390/ijms24108652)
Supplement: Supplementary file 1 [file ijms-24-08652-s001.zip › Table S2.docx]

Table S2 The basic information of WGS

| Strains | | 17978S | 17978R | A54R | A54S |
| --- | --- | --- | --- | --- | --- |
| Sequencing depth（×） | | 306.5438 | 280.3532 | 348.5033 | 214.7277 |
| Numbers of base pair（bp） | | 4075596 | 3917421 | 4236314 | 4108976 |
| GCcontent（%） | | 38.9 | 38.97 | 39.08 | 38.97 |
| Base pair of chromosomes（bp） | | 3901819 | 3892710 | 4056249 | 4029089 |
| Base pair of plasmids（bp） | Plasmid 1 | 148955 | - | 110967 | 10789 |
|  | Plasmid 2 | 24822 | 24711 | 69098 | 69098 |
| Number of coding genes | | 3933 | 3762 | 4159 | 4024 |
| Number of coding genes on chromosomes | | 3744 | 3732 | 3951 | 3923 |
| Number of coding genes on plasmids | | 189 | 30 | 208 | 101 |
| Number of tRNAs | | 73 | 73 | 75 | 74 |
| Number of rRNAs | | 18 | 18 | 18 | 18 |
