# Supplementary material for: The Mechanism of Tigecycline Resistance in Acinetobacter baumannii Revealed by Proteomic and Genomic Analysis
Source: Int J Mol Sci. 2023 May 12;24(10):8652. doi: 10.3390/ijms24108652 (PMC10218405; doi:10.3390/ijms24108652)
Supplement: Supplementary file 1 [file ijms-24-08652-s001.zip › Table S4.docx]

**Table S4** Single nucleotide variation(SNV) analysis of 17978R/S and A54R/S

| NO | SNV Pattern | | SNV Site | | | | Amino acid alternation | | Coding genes | | NO | | SNV Pattern | SNV Site | | | | Amino acid alternation | | | Coding genes |
| --- | --- | --- | --- | --- | --- | --- | --- | --- | --- | --- | --- | --- | --- | --- | --- | --- | --- | --- | --- | --- | --- |
|  |  |  | 17978S | | 17978R | |  |  |  |  |  |  |  | A54R | | | A54S |  |  |  |  |
| 1 | A-G | | 18814 | | 18803 | | noncoding region | | - | | 1 | | T-G | 1269504 | | | 1269495 | Glutamate-Alanine | | | putative protein |
| 2 | G-A | | 18816 | | 18804 | | noncoding region | | - | | 2 | | C-T | 1302967 | | | 1302958 | Threonine-Isoleucine | | | putative protein |
| 3 | G-A | | 19594 | | 19583 | | noncoding region | | - | | 3 | | G-A | 2847907 | | | 1567471 | synonymous | | | *ata* |
| 4 | G-A | | 21527 | | 21515 | | noncoding region | | - | | 4 | | A-G | 2847748 | | | 1567630 | synonymous | | | *ata* |
| 5 | A-G | | 21805 | | 21798 | | noncoding region | | - | | 5 | | A-C | 2847637 | | | 1567741 | synonymous | | | *ata* |
| 6 | A-G | | 21806 | | 21799 | | noncoding region | | - | | 6 | | A-G | 2847634 | | | 1567744 | synonymous | | | *ata* |
| 7 | A-G | | 22607 | | 22597 | | noncoding region | | - | | 7 | | C-T | 2847632 | | | 1567746 | synonymous | | | *ata* |
| 8 | G-A | | 22608 | | 22598 | | noncoding region | | - | | 8 | | G-A | 2847631 | | | 1567747 | Serine-Aspartic Acid | | | *ata* |
| 9 | A-C | | 23222 | | 23213 | | noncoding region | | - | | 9 | | A-G | 2847628 | | | 1567750 | synonymous | | | *ata* |
| 10 | G-T | | 377096 | | 378278 | | noncoding region | | - | | 10 | | G-A | 2846698 | | | 1568680 | synonymous | | | *ata* |
| 11 | C-G | | 445993 | | 447172 | | Lysine-Glycine | | *rplN* | | 11 | | A-G | 2846539 | | | 1568839 | synonymous | | | *ata* |
| 12 | G-T | | 453082 | | 454261 | | Glycine-Valine | | *rpoA* | | 12 | | A-C | 2846428 | | | 1568950 | synonymous | | | *ata* |
| 13 | A-C | | 526667 | | 527846 | | noncoding region | | - | | 13 | | A-G | 2846425 | | | 1568953 | synonymous | | | *ata* |
| 14 | C-T | | 662206 | | 663385 | | Lysine-cysteine | | ACX60_03215 | | 14 | | C-T | 2846423 | | | 1568955 | synonymous | | | *ata* |
| 15 | T-C | | 837538 | | 838717 | | noncoding region | | - | | 15 | | G-A | 2846422 | | | 1568956 | Serine-Aspartic Acid | | | *ata* |
| 16 | G-A | | 1929554 | | 1919161 | | Alanine-Valine | | *srrA* | | 16 | | A-G | 2846419 | | | 1568959 | synonymous | | | *ata* |
| 17 | C-A | | 2385973 | | 2375576 | | noncoding region | | - | | 17 | | G-A | 2756449 | | | 1658929 | Threonine-Isoleucine | | | putative protein |
| 18 | | T-C | | 3006160 | | 2996034 | | synonymous | | *tuf* | | 18 | A-G | | 2239282 | 2177285 | | | synonymous | ABZJ_02130 | |
| 19 | | G-A | | 3140028 | | 3129902 | | synonymous | | *frpC* | | 19 | T-C | | 2164680 | 2251887 | | | synonymous | ABZJ_02068 | |
| 20 | | T-C | | 3587232 | | 3577088 | | synonymous | | *tuf* | | 20 | T-C | | 1755433 | 2658089 | | | synonymous | *ahpF* | |
|  | |  | |  | |  | |  | |  | | 21 | C-A | | 1755373 | 2658149 | | | synonymous | *ahpF* | |
|  | |  | |  | |  | |  | |  | | 22 | G-A | | 1755370 | 2658152 | | | synonymous | *ahpF* | |
|  | |  | |  | |  | |  | |  | | 23 | T-C | | 1755343 | 2658179 | | | synonymous | *ahpF* | |
|  | |  | |  | |  | |  | |  | | 24 | C-T | | 1755334 | 2658188 | | | synonymous | *ahpF* | |
|  | |  | |  | |  | |  | |  | | 25 | G-A | | 1430518 | 2983004 | | | synonymous | *ahpF* | |
|  | |  | |  | |  | |  | |  | | 26 | A-G | | 1430509 | 2983013 | | | synonymous | *ahpF* | |
|  | |  | |  | |  | |  | |  | | 27 | C-T | | 1430482 | 2983040 | | | synonymous | *ahpF* | |
|  | |  | |  | |  | |  | |  | | 28 | G-T | | 1430479 | 2983043 | | | synonymous | *ahpF* | |
|  | |  | |  | |  | |  | |  | | 29 | A-G | | 1430419 | 2983103 | | | synonymous | *ahpF* | |
|  | |  | |  | |  | |  | |  | | 30 | A-G | | 3148953 | 3121793 | | | synonymous | *tuf* | |
|  | |  | |  | |  | |  | |  | | 31 | A-G | | 3741557 | 3714397 | | | synonymous | *tuf* | |
