# Supplementary material for: The Mechanism of Tigecycline Resistance in Acinetobacter baumannii Revealed by Proteomic and Genomic Analysis
Source: Int J Mol Sci. 2023 May 12;24(10):8652. doi: 10.3390/ijms24108652 (PMC10218405; doi:10.3390/ijms24108652)
Supplement: Supplementary file 1 [file ijms-24-08652-s001.zip › Table S5.docx]

| category |  | 17978S | 17978R | A54R | A54S |
| --- | --- | --- | --- | --- | --- |
| resistance genes |  | 61 | 60 | 82 | 73 |
| virulence factors | setA | 220 | 216 | 219 | 217 |
|  | setB | 256 | 251 | 256 | 254 |
| genome islands |  | 3 | 3 | 5 | 4 |

Table S5 Antibiotic resistance genes, virulence factors and genome islands in the genomes
