# Supplementary figures and images for: The Mechanism of Tigecycline Resistance in Acinetobacter baumannii Revealed by Proteomic and Genomic Analysis
Source: Int J Mol Sci. 2023 May 12;24(10):8652. doi: 10.3390/ijms24108652 (PMC10218405; doi:10.3390/ijms24108652)

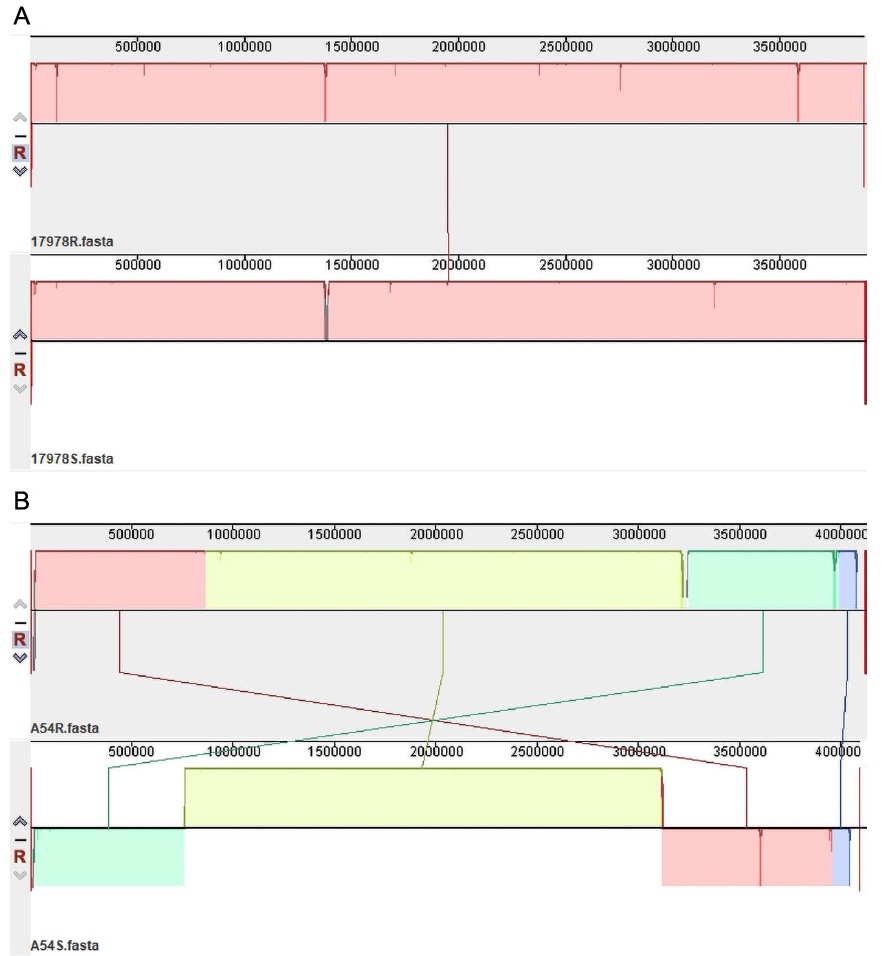

Supplement: Supplementary file 1 [file ijms-24-08652-s001.zip › Figure S1 Genome collinearity of 17978S and 17978R (A), and A54R and A54S (B) with MAUVE.tif]
